# Oxaliplatin Causes Transient Changes in TRPM8 Channel Activity

**DOI:** 10.3390/ijms22094962

**Published:** 2021-05-07

**Authors:** Vittoria Rimola, Tabea Osthues, Vanessa Königs, Gerd Geißlinger, Marco Sisignano

**Affiliations:** 1 Institute of Clinical Pharmacology, Pharmazentrum Frankfurt/ZAFES, University Hospital, Goethe-University, 60590 Frankfurt am Main, Germany; rimola@med.uni-frankfurt.de (V.R.); geisslinger@em.uni-frankfurt.de (G.G.); 2 Fraunhofer Institute for Translational Medicine and Pharmacology ITMP, Theodor-Stern-Kai 7, 60596 Frankfurt am Main, Germany; tabea.osthues@itmp.fraunhofer.de (T.O.); Vanessa.Koenigs@itmp.fraunhofer.de (V.K.)

**Keywords:** oxaliplatin, TRPM8, desensitization, acute pain, neuropathic pain

## Abstract

Oxaliplatin is a third-generation platinum-based anticancer drug that is widely used as first-line treatment for colorectal carcinoma. Patients treated with oxaliplatin develop an acute peripheral pain several hours after treatment, mostly characterized by cold allodynia as well as a long-term chronic neuropathy. These two phenomena seem to be causally connected. However, the underlying mechanisms that trigger the acute peripheral pain are still poorly understood. Here we show that the activity of the transient receptor potential melastatin 8 (TRPM8) channel but not the activity of any other member of the TRP channel family is transiently increased 1 h after oxaliplatin treatment and decreased 24 h after oxaliplatin treatment. Mechanistically, this is connected with activation of the phospholipase C (PLC) pathway and depletion of phosphatidylinositol 4,5-bisphosphate (PIP_2_) after oxaliplatin treatment. Inhibition of the PLC pathway can reverse the decreased TRPM8 activity as well as the decreased PIP_2_-concentrations after oxaliplatin treatment. In summary, these results point out transient changes in TRPM8 activity early after oxaliplatin treatment and a later occurring TRPM8 channel desensitization in primary sensory neurons. These mechanisms may explain the transient cold allodynia after oxaliplatin treatment and highlight an important role of TRPM8 in oxaliplatin-induced acute and neuropathic pain.

## 1. Introduction

Oxaliplatin is a first-line cytostatic and the major component of the widely used FOLFOX regimen for the treatment of advanced colorectal cancer [1]. However, in up to 90% of the treated patients oxaliplatin causes neurotoxicity and acute pain that already begins several hours after treatment and that is characterized by cold allodynia [2,3]. Apart from these transient effects, in up to 80% of the patients oxaliplatin also causes a long-term distal sensory neuropathy that can persist lifelong [4,5]. The acute pain after oxaliplatin treatment usually subsides within 2–3 days after treatment; however, clinical studies suggest a causal connection of the oxaliplatin-induced acute pain and the later occurring chronic neuropathy [6]. Although many substances were investigated in clinical trials, currently, there is no preventative treatment available for oxaliplatin-induced neuropathic pain [7].

The cellular and molecular mechanisms of the acute response to oxaliplatin are still unclear. Several studies suggest that oxaliplatin treatment rapidly causes damage to the peripheral nervous system by inducing synthesis of reactive oxygen species (ROS), mitochondrial dysfunction and altered activity of neuronal ion channels [8,9,10,11].

The formation of ROS had already been suggested as target for chemotherapy-induced neuropathic pain (CINP) before. However, clinical studies using antioxidants or neuroprotective substances to prevent or treat chemotherapy-induced neuropathic pain, such as vitamin E, glutathione or α-lipoic acid, all failed to ameliorate CINP in patients [12].

Altered activity of ion channels has been reported to contribute to oxaliplatin-induced neuropathic pain. In this context, the activity of the voltage-gated sodium channel Na_(v)_1.6, the potassium channels KCNK2 and KCNK4 as well as the activity of transient receptor potential (TRP) channels was found to be altered by oxaliplatin [11,13,14].

TRP channels are ligand-gated ion channels. Some members of this ion channel family can detect external noxious stimuli, such as heat, cold and chemicals. They respond to these stimuli by opening their pores and allowing calcium to enter the cell [15]. Several TRP channels are highly expressed in sensory neurons. Upon their activation and subsequent calcium influx, depolarization and action potentials can be triggered, causing acute or persistent pain [16]. The most important TRP channels for pain are TRPV1 (vanilloid), TRPA1 (ankyrin) and TRPM8 (melastatin). TRPV1 can be activated by noxious heat or capsaicin, TRPA1 by pungent environmental substances and TRPM8 by noxious cold or menthol [16]. More recently, TRPM3 was identified as an additional receptor for noxious heat [17,18].

Altered activity of TRPA1, TRPV1 and TRMP8 have been attributed to oxaliplatin-induced neuropathic pain in previous preclinical studies [13]. In these studies, the TRP-channel activity was assessed several days to weeks after initial oxaliplatin-treatment, thus investigating its contribution to oxaliplatin-induced long-term chronic neuropathic pain. However, as shown recently, the early oxaliplatin-induced acute pain, which starts several hours after treatment in patients, is causally linked to the chronic neuropathy, and patients with strong oxaliplatin-induced acute pain have a higher risk of developing a more severe chronic neuropathy [6]. It is therefore necessary to investigate TRP-channel activity at early timepoints after oxaliplatin treatment to understand its contribution to oxaliplatin-induced neuropathy. Unfortunately, this has not yet been addressed experimentally.

Here, we investigate the activity of the ligand-gated calcium channels TRPV1, TRPA1, TRM8, TRPM3 and the purinergic receptor P2X3, a nucleotide-gated calcium-channel that is expressed in sensory neurons and that is known to contribute to chronic pain [19]. We found that the activity of TRPM8 but not of the other calcium channels was transiently increased 1 h after oxaliplatin treatment and decreased 24 h after oxaliplatin treatment, a timepoint at which mice show mechanical hypersensitivity [20]. This altered TRPM8 activity was not related to changes in the channel‘s gene expression. Instead, we found that the decreased TRPM8 activity 24 h after oxaliplatin treatment was mediated by phospholipase C (PLC) and phosphatidylinositol 4,5-bisphosphate (PIP_2_)- depletion. In summary, we found that aberrant TRPM8 activity in sensory neurons was a crucial and a very early effect of oxaliplatin-treatment, an effect that seems to be linked to oxaliplatin-induced neuropathy and that may be targeted pharmacologically to alleviate oxaliplatin-induced pain.

## 2. Results

### 2.1. The TRPM8 Channel Activity Is Affected after Oxaliplatin-Induced Acute Peripheral Pain

**Figure 1:**
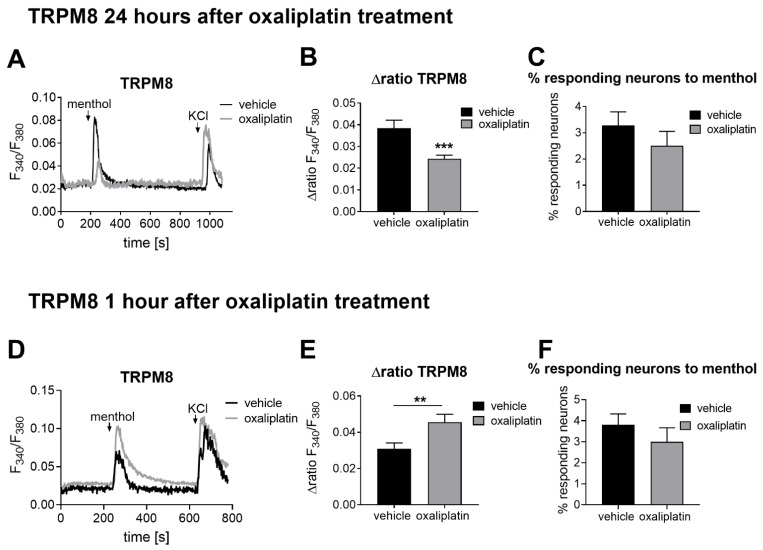
TRPM8 channel activity and number of neurons responding to TRPM8 channel specific agonists 24 h and 1 h after oxaliplatin (3 mg/kg) or vehicle treatment. (**A**,**D**) Representative Ca^2+^ responses after TRPM8 channel stimulation with menthol after (**A**) 24 h (menthol 100 µM, 30 s) or (**D**) 1 h (menthol 300 µM, 45 s) after oxaliplatin treatment. (**B**,**E**) Δratio F_340_/F_380_ of the amplitude after a transient Ca^2+^ influx after TRPM8 channel stimulation (**B**) 24 h or (**E**) 1 h after oxaliplatin treatment. Data represent the means ± SEM from *n* = 24–64 primary sensory neurons per condition from *n* = 2–5 mice; ** *p* < 0.01; *** *p* < 0.001; unpaired two-tailed *t*-test with Welch’s correction. (**C**,**F**) Percentage of neurons responding to TRPM8 (**C**) 24 h or (**F**) 1 h after oxaliplatin treatment. Data represent the means ± SEM from *n* = 20–42 independent measurements per condition; unpaired two-tailed *t*-test with Welch’s correction. Primary sensory neurons were identified by stimulating the DRG cultures with 50 mM KCl for 60 s.

Previously, we showed that oxaliplatin induced mechanical hypersensitivity 24 h after treatment [20]. For the current study, we first investigated whether the activity of the TRPM8 channel was altered in response to oxaliplatin treatment. Using the TRPM8 specific agonist menthol (100 µM, 30 s), we observed that the TRPM8 channel activity was significantly reduced in oxaliplatin-treated animals after 24 h when compared with vehicle-treated animals (*p* = 0.0006, Figure 1A,B). Similar to the other investigated channels, the number of neurons responding to the selective TRPM8 agonist menthol was not affected (Figure 1C).

To investigate the impact of oxaliplatin on the TRPM8 channel more transiently, we next analyzed TRPM8 activity 1 h after oxaliplatin treatment. Interestingly, oxaliplatin treatment led to significantly increased TRPM8 channel activity when compared with vehicle-treated animals (*p* = 0.0089, Figure 1D,E). Likewise, we analyzed the number of neurons responding to the TRPM8 channel agonist menthol 1 h after oxaliplatin treatment (Figure 1F). As already observed for the later timepoint, no alterations in the number of responding neurons could be observed 1 h after oxaliplatin treatment (Figure 1C,F).

**Figure 2:**
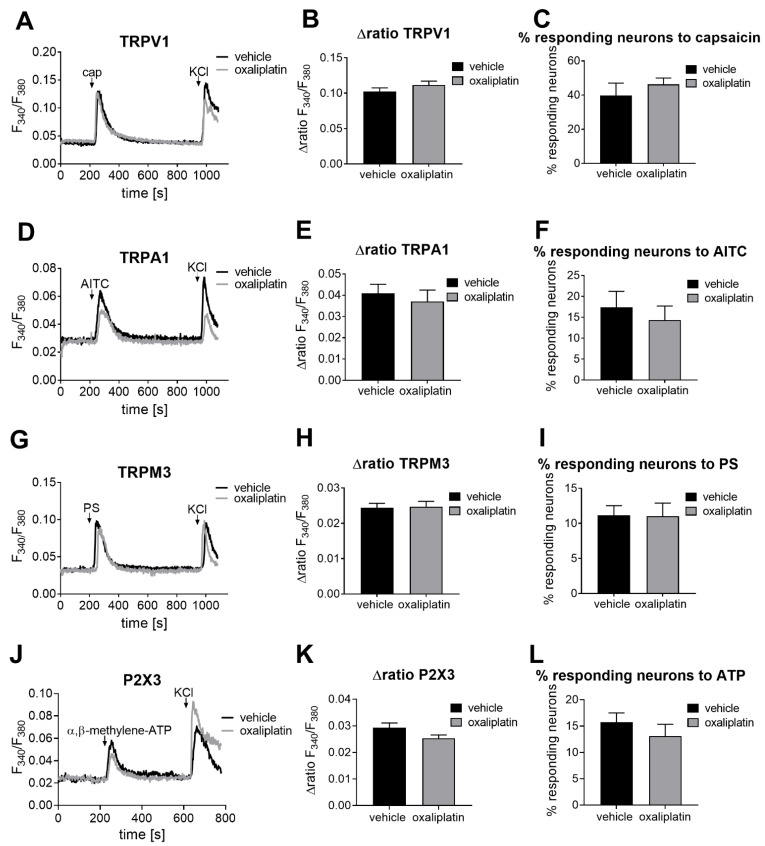
TRPV1, TRPA1, TRPM3 and P2X3 channel activity and number of neurons responding to their selective agonists 24 h after oxaliplatin (3 mg/kg) or vehicle treatment. (**A**,**D**,**G,J**) Representative Ca^2+^ responses after (**A**) TRPV1, (**D**) TRPA1, (**G**) TRPM3, or (**J**) P2X3 channel stimulation with their corresponding agonists ((**A**) TRPV1: cap (capsaicin) 200 nM, 20 s; (**D**) TRPA1: AITC (allyl isocyanate) 100 µM, 30 s; (**G**) TRPM3: PS (pregnenolone sulfate) 40 µM, 45 s; (**J**) P2X3: α,β-methylene-ATP 50 µM, 20 s). (**B**,**E**,**H**,**K**) Δratio F_340_/F_380_ of the amplitude after a transient Ca^2+^ influx after (**B**) TRPV1, (**E**) TRPA1, (**H**) TRPM3 or (**K**) P2X3 channel stimulation. Data represent the means ± SEM from *n* = 45–89 primary sensory neurons per condition from *n* = 2–3 mice; unpaired two-tailed *t*-test with Welch´s correction. (**C**,**F**,**I**,**L**) Percentage of neurons responding to (**C**) TRPV1, (**F**) TRPA1, (**I**) TRPM3, or (**L**) P2X3 channel agonists. Data represent the means ± SEM from *n* = 4–18 independent measurements per condition; unpaired two-tailed *t*-test with Welch´s correction. Neurons were identified by stimulating the DRG cultures with 50 mM KCl for 60 s.

We next investigated the activity as well as the number of responding neurons of four additional ligand-gated calcium channels that have previously been suggested to contribute to persistent pain states and to hypersensitivity after oxaliplatin treatment (Figure 2). We first started to investigate the TRPV1 and the TRPA1 channel activity as well as the number of neurons responding to their agonists capsaicin (200 nM, 20 s) and AITC (allyl isocyanate; 100 µM, 30 s), as several studies described an involvement of both channels in the induction of oxaliplatin-induced acute peripheral pain [14,21,22]. However, neither TRPV1 nor TRPA1 channel activity nor the number of responding neurons was affected 24 h after oxaliplatin treatment (Figure 2A–F). Viable neurons were identified by a short KCl stimulation at the end of each measurement, causing depolarization and activation of voltage-gated calcium channels. Previously, we could observe that the mRNA expressions of TRPV1 and TRPA1 were unchanged after oxaliplatin treatment [20]. We next investigated the mRNA expression levels of several other ion channels that have previously been connected with oxaliplatin-induced hypersensitivity [14]. For this experiment, DRGs were harvested from mice 24 h after oxaliplatin treatment (3 mg kg^−1^), and qPCR analysis was performed. However, we could not observe any alterations in mRNA expression levels for KCNK2, KCNK4, HCN1, piezo1 and P2X3 24 h after oxaliplatin treatment (Figure S1). Interestingly, the TRPM3 transcript was significantly increased in oxaliplatin-treated mice when compared with vehicle-treated animals after 24 h (*p* = 0.0348, Figure S1). We next investigated the activity of the TRPM3 channel but could not detect any differences in the activity and number of neurons responding to the TRPM3-specific agonist pregnenolone sulfate (PS, 40 µM, 45 s, Figure 2G–I) [23]. We additionally analyzed the activity of the nucleotide-gated P2X3 channel and the number of neurons responding to the P2X3-specific agonist α,β-methylene-ATP (50 µM, 20 s) [24] because ATP-dysregulation after oxaliplatin treatment was described as a mechanism of oxaliplatin-induced pain by several studies [9,25]. However, when compared with vehicle-treated mice, no differences in Ca^2+^ influx in primary sensory neurons nor in the number of neurons responding to α,β-methylene-ATP could be detected after stimulating the P2X3 ion channel of oxaliplatin-treated animals (Figure 2J–L).

In summary, measuring the ligand-gated and calcium-permeable ion-channels revealed transient changes of TRPM8 activity in sensory neurons 1 h and 24 h after oxaliplatin treatment.

### 2.2. The TRPM8 Channel Activity Can Be Reconstituted after PLC and PKC Pathway Inhibiton

**Figure 3:**
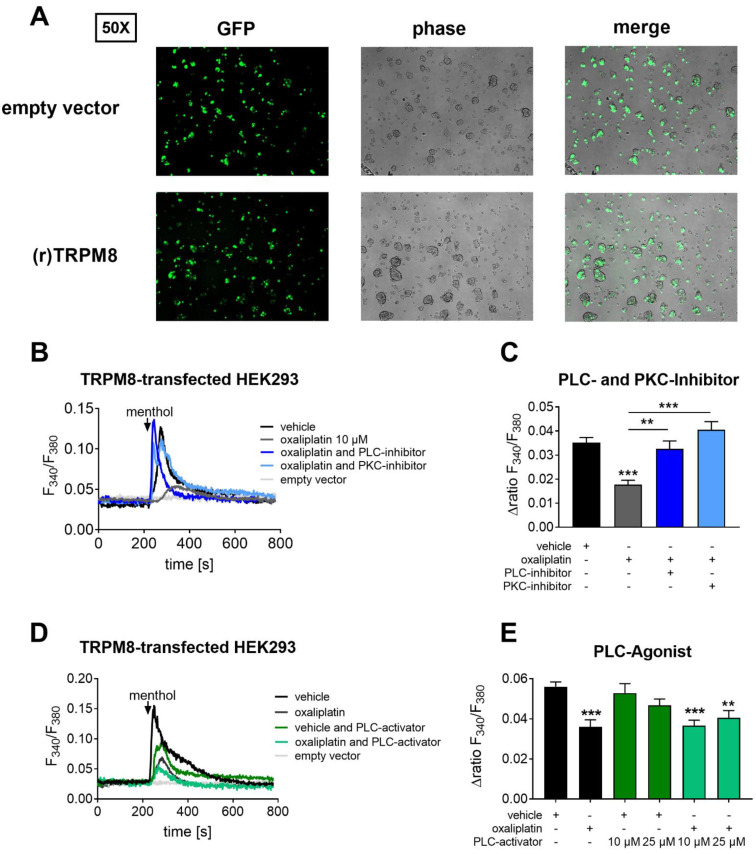
Transfected HEK cells show a decreased TRPM8 channel activity that can be reconstituted by PLC and PKC pathway inhibition. (**A**) Representative fluorescence and phase contrast images of TRPM8 and empty-vector (pEGFP)-transfected HEK cells captured at 50× magnification. (**B**) Representative Ca^2+^ responses after menthol stimulation (300 µM, 45 s) of TRPM8 (dark grey) and empty-vector-transfected (light grey) HEK cells that were treated with vehicle (0.052% *v/v* DMSO), 10 µM oxaliplatin (grey), ±1 µM of the PLC inhibitor U 73122 (blue), ±1 µM of the PKC inhibitor GF 109203X (light blue) for 24 h. (**C**) Δratio F_340_/F_380_ of the amplitude after a transient Ca^2+^ influx after stimulating TRPM8-transfected HEK cells. Data represent the means ± SEM from *n* = 44–152 cells per condition; ** *p* < 0.01; *** *p* < 0.001; one-way ANOVA and Dunnett´s post hoc test. (**D**) Representative Ca^2+^ responses after menthol stimulation (300 µM, 45 s) of TRPM8-transfected (dark grey) and empty-vector-transfected (light grey) HEK cells treated with vehicle (0.05% *v/v* DMSO), TRPM8-transfected HEK cells treated with 10 µM oxaliplatin (grey), 10 µM oxaliplatin ±10 µM (green) or 25 µM (light green) of the PLC activator *m*-3M3FBS for 24 h. (**E**) Δratio F_340_/F_380_ of the amplitude after a transient Ca^2+^ influx after stimulating TRPM8-transfected HEK cells. Data represent the means ± SEM from *n* = 62–160 TRPM8-transfected HEK cells per condition; ** *p* < 0.01; *** *p* < 0.001; one-way ANOVA and Dunnett´s post hoc test.

Since we saw transient changes in the TRPM8 channel activity after oxaliplatin treatment and a decrease of TRPM8 activity at the later timepoint, we next tested the hypothesis that the TRPM8 channel is desensitized after oxaliplatin treatment. It was shown previously that TRPM8 channel desensitization can occur after activation of the phospholipase C (PLC) pathway followed by depletion of PIP_2_ (phosphatidylinositol 4,5-bisphosphate) to IP_3_ (inositol 1,4,5-triphosphate) and DAG (1,2-diacylglycerol) [26]. To investigate the influence of oxaliplatin, we used a heterologous expression system with TRPM8 transfected HEK293 cells since the TRPM8 channel is only expressed in 5–10% of primary sensory neurons, and the downstream signaling of TRPM8 activation is triggered only in this fraction of neurons (Figure 3A). It was important to check whether the heterologous expression system with TRPM8-transfected HEK cells reflects the findings of the Ca^2+^ imaging measurements with DRG neurons. We therefore treated TRPM8-transfected HEK cells with two different oxaliplatin concentrations (5 µM and 10 µM) for 24 h prior to Ca^2+^ imaging measurements. Treating the cells with 5 µM showed no effect (Figure S2). However, treatment with 10 µM oxaliplatin for 24 h showed a significantly reduced TRPM8 channel activity similar to the effect in DRG neurons (*p* < 0.0001, Figure 3B,C). To ensure that the heterologous expression system was comparable to the ex vivo system with primary sensory neurons, we also investigated the activity of the TRPV1 channel in TRPV1-transfected HEK cells (Figure S3). TRPV1 activity was not affected by oxaliplatin treatment in the heterologous expression system (Figure S3), which is in accordance with our previous results in primary sensory neurons 24 h after oxaliplatin treatment (Figure 2A,B). We conclude that the heterologous expression system can appropriately reflect the activity of the TRPV1 and the TRPM8 channel and can do so in a comparable manner to its activation in primary sensory neurons 24 h after oxaliplatin treatment.

Next, we analyzed the underlying mechanisms that lead to TRPM8 channel desensitization after oxaliplatin treatment. We therefore performed Ca^2+^ imaging with TRPM8-transfected HEK cells that were treated with a PLC inhibitor (U 73122 [27], 1 µM for 24 h) in addition to oxaliplatin. Interestingly, the decreased TRPM8 channel activity in transfected HEK cells could be reversed after PLC inhibition (*p* = 0.0073, Figure 3B).

It is known that PLC pathway activation causes hydrolyzation of PIP_2_ into IP_3_ and DAG. This can also lead to the activation of the protein kinase C (PKC). As previously shown, PKC activation may lead to TRPM8 channel desensitization as well [28]. We analyzed TRPM8 channel activity after inhibiting PKC (Figure 3B,C) and, similar to PLC pathway inhibition, we could observe significantly increased TRPM8 channel activity in transfected HEK cells after PKC inhibition (GF 109203X [29], 1 µM for 24 h, *p* < 0.0001). These results suggest that acute oxaliplatin treatment can induce PLC and PKC pathway activation.

To determine the effect of the PLC–PKC pathway in modulating TRPM8 activity, we next used a specific PLC agonist (Figure 3D). While we saw a tendency of decreased TRPM8 activity with the PLC activator alone (*m*-3M3FBS 25 µM), we could reproduce the reduction of TRPM8 activity by oxaliplatin (oxaliplatin: *p* = 0.003; oxaliplatin ±10 µM PLC agonist: *p* < 0.0001; oxaliplatin ±25 µM PLC agonist: *p* < 0.0019). Additional treatment with the PLC activator (*m*-3M3FBS [30], 10 µM and 25 µM for 24 h) did not cause additional reduction of TRPM8 activity, indicating that the desensitizing effect of oxaliplatin on TRPM8 activity may already be at maximum and cannot be further potentiated by additional PLC activation (Figure 3D,E).

### 2.3. TRPM8 Channel Desensitization Occurs after TRPM8 Channel Activation and PIP_2_ Depletion upon PLC Pathway Activation

**Figure 4:**
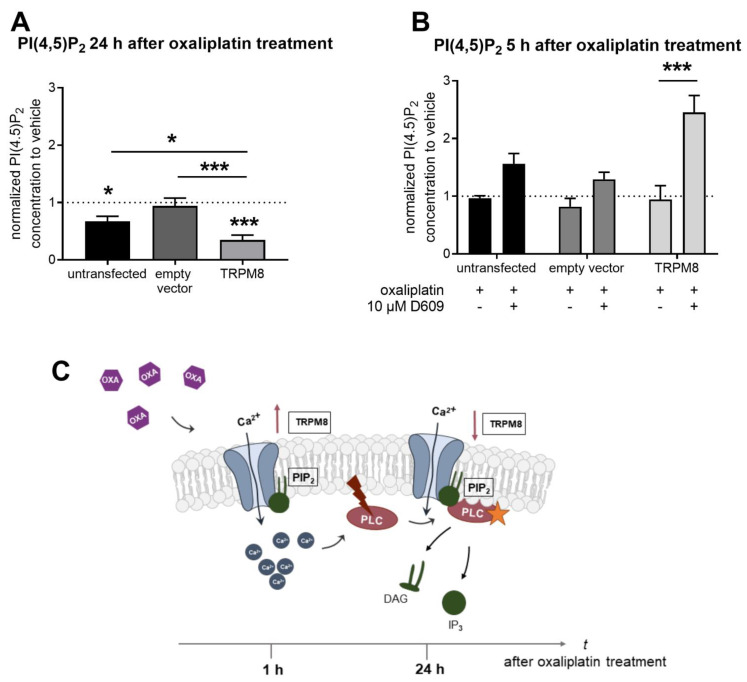
Phosphatidyl 4,5-bisphosphate concentrations are altered after oxaliplatin treatment. (**A**) Relative PIP_2_ (phosphatidylinositol 4,5-bisphosphate) concentrations of untransfected HEK cells, empty-vector-transfected and TRPM8-transfected HEK cells treated with vehicle (saline) or 10 µM oxaliplatin for 24 h. Data represent the means ± SEM from *n* = 10–12 dishes per condition. * *p* < 0.05; *** *p* < 0.001; one-way ANOVA and Dunnett´s multiple comparison test. (**B**) Relative PIP_2_ concentrations of untransfected HEK cells, empty-vector-transfected and TRPM8-transfected HEK cells that were treated with vehicle (saline), 10 µM oxaliplatin ±10 µM of the PLC inhibitor D609 for 5 h. Data represent the means ± SEM from *n* = 4–6 dishes per condition. *** *p* < 0.001; one-way ANOVA and Dunnett´s multiple comparison test. (**C**) Schematic overview of the proposed mechanisms leading to TRPM8 channel desensitization after acute oxaliplatin treatment. TRPM8 channel activation causes PLC pathway activation. In turn, PLC hydrolyzes PIP_2_ to IP_3_ (inositol 1,4,5-triphosphate) and DAG (1,2-diacylglycerol). PIP_2_-depletion is responsible for TRPM8 channel desensitization after oxaliplatin treatment.

In the previous experiments, we observed that PLC pathway inhibition can reverse TRPM8 channel desensitization (Figure 3B,C). Since PLC activation leads to a depletion of its substrate PIP_2_, we next analyzed the PIP_2_ concentrations of untransfected, empty-vector-transfected and TRPM8-transfected HEK cells in the presence of oxaliplatin using a PIP_2_ ELISA. Comparing the relative PIP_2_ concentrations of vehicle- and oxaliplatin-treated HEK cells, we saw a significantly reduced PIP_2_ concentration, especially in TPM8-transfected HEK cells (*p* < 0.0001, Figure 4A). Interestingly, the relative PIP_2_ concentrations in TRPM8-transfected HEK cells were significantly decreased after oxaliplatin treatment in comparison with the empty-vector-transfected HEK cells and untransfected HEK cells (TRPM8-transfected HEK cells vs. empty-vector-transfected HEK cells after oxaliplatin treatment: *p* = 0.0001; TRPM8-transfected HEK cells vs. untransfected HEK cells after oxaliplatin treatment: *p* = 0.0499, Figure 4A). These results show that oxaliplatin can lead to PIP_2_ depletion.

To verify the effect of oxaliplatin activation of the PLC pathway leading to PIP_2_ depletion, we next performed a PIP_2_ ELISA with HEK cells that were treated with vehicle, oxaliplatin and a PLC inhibitor (D609 [31], 10 µM for 5 h, Figure 4B). The ELISA measurements revealed a significantly increased PIP_2_ concentration in TRPM8-transfected HEK cells after treatment with the PLC inhibitor in addition to oxaliplatin, when compared with TRPM8-transfected HEK cells treated with oxaliplatin alone (*p* < 0.0001, Figure 4B). The results obtained from the PIP_2_ ELISA strengthen the presumption that oxaliplatin can affect TRPM8 channel activity by PLC pathway activation.

As shown in Figure 4C, based on the results of this study, we propose that TRPM8 channel desensitization after oxaliplatin treatment (24 h) occurs due to early TRPM8 channel activation and subsequent PLC pathway activation. In turn, PLC can degrade PIP_2_, which positively regulates TRPM8 channel activity, into IP_3_ and DAG. Eventually, activation of the PLC pathway can cause TRPM8 channel desensitization after oxaliplatin treatment (Figure 4C).

## 3. Discussion

In summary, we could observe that among the different investigated calcium channels, only TRPM8 was affected by oxaliplatin treatment for 24 h. The decreased TRPM8 channel activity is likely to occur due to an early transient activation and subsequent PLC (phospholipase C) pathway activation. Oxaliplatin caused decreases in PIP_2_ (phosphatidylinositol 4,5-bisphosphate) concentrations, which were observed mainly in TRPM8-transfected HEK cells, and this effect could be reversed with a specific PLC inhibitor. These findings suggest a transient and dynamic change of TRPM8 activity after oxaliplatin treatment.

Oxaliplatin does not directly activate the TRPM8 channel [14]. This leads to the conclusion that modulation of TRPM8 by oxaliplatin is mediated in an indirect manner. It was previously described that oxaliplatin increases the expression of TRPM8 in medium-sized neurons of the dorsal root ganglia four days after treatment [32]. In another study, the authors did not see any differences in TRPM8 expression 90 h after treatment [14].

The effects of PLC are crucial for the activity of multiple ligand-gated calcium channels, among them several thermo-sensitive TRP channels. The substrate of PLC, PIP_2_, was previously shown to modulate TRP channel activity with contrasting effects. For example, PIP_2_ can increase the activity of TRPM5, is required for osmotic activation of TRPV4, and can inhibit desensitization of TRPA1, while it can decrease the activity of TRPV3 [33].

For TRPM8, it was shown that PIP_2_ seems to bind to a C-terminal region, causing positive modulation of the channel [26]. Among the PLC isoforms, the family of PLCds and the isoform PLCd4 seem to be responsible for modulating the activity of TRPM8 [34,35]. The crucial role of PIP_2_ for normal TRPM8 activity was confirmed by incorporation of TRPM8 in lipid bilayers. In this system, both menthol and cold activation of TRPM8 depended on PIP_2_ [36]. Likewise, it was shown that PIP_2_ depletion can lead to tachyphylaxis of TRPM8 and that PIP_2_ is required for maintaining the threshold temperature of TRPM8 [37,38], which is in line with our data.

For TRPA1, another supposed cold receptor, the activity modulation of PIP_2_ seems to be different and more complex. For example, PIP_2_ was shown to inhibit mustard oil-induced desensitization of TRPA1 and TRPV1-dependent cross-desensitization of TRPA1 in cells expressing both channels [39,40]. However, there are contrasting reports indicating negligible effects of PIP_2_ on TRPA1 activity [41,42].

According to our results, TRPM8 activity is increased after one hour of oxaliplatin treatment. Although it is known that oxaliplatin does not directly activate TRPM8 [14], it has been shown before that oxaliplatin can rapidly enter sensory neurons and gilal cells after treatment, probably via organic cation transporters (OCT), and can remain in these cells for a long time [43,44]. The accumulation of oxaliplatin in neurons may lead to increased TRPM8 activity, which is in accordance with the acute cold pain that is observed rapidly after treatment in patients [6].

In contrast, after 24 h, TRPM8 activity was decreased, which is probably mediated by previous calcium influx, leading to PLC activation and PIP_2_ depletion. It remains to be investigated whether the oxaliplatin levels within the neurons remain the same after initial uptake or if they increase slowly. Moreover, the metabolization of oxaliplatin into oxalate and platinum may additionally modulate TRPM8 activity by accumulation of oxalate and calcium depletion in the cytosol [45]. While the initial increase in TRPM8 activity fits to the acute cold pain response in patients, the decreased TRPM8 activity after 24 h seems to diverge.

However, we suggest that TRPM8 is the first responder to oxaliplatin accumulation in sensory neurons. At later timepoints, this system may be exhausted and other stress responses may take over to maintain the neuron in a sensitized state. After three to four days, the expressions of TRPM8, KCNK2, KCNK4, TRPA1 and Na_v_1.8 increase in sensory neurons to initiate a new phase of increased neuronal activity and oxaliplatin-induced hypersensitivity [14,32].

In this regard, it would be interesting to investigate whether an early blockade of excessive TRPM8 activity can also prevent subsequent events that lead to chronic neuropathic pain.

While we see an increased expression of the TRPM3 transcript after oxaliplatin treatment, we do not see any detectable increase of TRPM3 activity in our calcium imaging experiments. We assume that oxaliplatin causes several transcriptional stress responses in sensory neurons, among them the early increase of TRPM3 expression. However, due to posttranscriptional or posttranslational modifications, increased expression does not necessarily correlate with increased protein synthesis or activity. Moreover, several alternative splice variants of TRPM3 have been described that may alter TRPM3 activity and make it more difficult to compare the expression of the channel with its activity [46]. In summary, while TRPM3 represents an important calcium channel that is relevant for pathological pain states, from our observations in this manuscript, we do not have any evidence for its contribution to oxaliplatin-induced acute pain.

Transient changes in TRPM8 channel activity may play an important role in the development of cold allodynia in oxaliplatin-induced acute peripheral pain as shown by oxaliplatin studies focusing on acutely induced pain. Even though we could not observe any changes in the activity of the other TRP channels, PIP_2_ is known to modulate the activity of several other TRP channels [47]. For example, it was shown that PIP_2_ can lead to TRPV1 channel sensitization [48]. Moreover, Anand et al., reported an increased responsiveness of the TRPV1 channel after acute exposure of oxaliplatin to DRG neurons [49]. According to our findings, apart from PIP_2_ depletion, PKC activation can also cause TRPM8 desensitization. A similar mechanism has been observed before by Premkumar et al., who showed that PKC activation can result in dephosphorylation of TRPM8 after bradykinin stimulation, causing decreased TRPM8 activity [50]. Interestingly, PKC seems to have contrasting effects on the activity of different TRP channels. It can mediate dephosphorylation of TRPM8 and reduce its activity, but for example, in TRPV1-positive neurons, it causes phosphorylation of TRPV1 and increased activity of the channel [51]. These contrasting mechanisms of both PLC and PKC strengthen the presumption that heat and cold nociceptors are regulated in a reciprocal manner and that the activation of one of the two neuronal populations leads to decreased activity of the other population, allowing it to focus on relevant external stimuli.

Nevertheless, we could show that particularly the activity of the TRPM8 channel is affected during oxaliplatin-induced acute peripheral pain. This is in line with the findings of previous studies that showed an involvement of the TRPM8 channel during oxaliplatin-induced neuropathic pain but which investigated later timepoints after oxaliplatin treatment. We conclude that targeting TRPM8 early after oxaliplatin treatment may ameliorate oxaliplatin-induced acute pain and may thus reduce the risk of patients developing a strong long-term neuropathy.

## 4. Materials and Methods

### 4.1. Animals

All animal experiments were approved by the local Ethics Committees for Animal Research (Darmstadt, Germany) under the approval code FK/1113, approved on 29 March 2019. In addition, all animal experiments were performed according to the Working Group PPRECISE (Preclinical Pain Research Consortium for Investigating Safety and Efficacy) [52] and the recommendation of the Guide of the Care and Use of Laboratory Animals of the National Institute of Health and the ARRIVE guidelines [53].

All experimental C57BL/6NRj animals were purchased from the commercial breeding company Janvier (Le Genest-Saint-Isle, France). They were housed in a day night cycle of a 12 h rhythm and food and water were available ad libitum. In addition, all animal experiments were performed with 8- to 12-week-old C57BL/6NRj male mice.

### 4.2. Oxaliplatin-Induced Acute Peripheral Pain Model

First, an oxaliplatin stock (Cayman Chemical, Ann Arbor, MI, USA) solution of 3 mg/mL in autoclaved deionized water was prepared. Then, the oxaliplatin stock solution of 3 mg/mL was diluted 1:4 in saline (sodium chloride 0.9% (*v/v*); Fresenius Kabi, Bad Homburg, Germany). Finally, a dose of 3 mg/kg of the 1:4 diluted oxaliplatin stock solution or vehicle (saline; 0.9% sodium chloride) was injected intraperitoneally (i.p.) in 8- to 12-week-old C57BL/6NRj male animals as described previously [22]. Thus, the application of the described oxaliplatin-induced neuropathic pain model was in accordance with the suggestions of the PPRECISE Working Group [52].

### 4.3. Animal Tissue Isolation

For qPCR analysis, dorsal root ganglia (DRGs) of oxaliplatin- and vehicle-treated mice after 24 h were used. For Ca^2+^ imaging experiments, DRGs of oxaliplatin- and vehicle-treated animals after 1 h and 24 h were used.

DRG tissue dissection from oxaliplatin- and vehicle-treated male mice was performed as previously described [54].

DRG tissue for qPCR analysis was immediately frozen in liquid nitrogen and stored until RNA-isolation at −80 °C. For Ca^2+^ imaging experiments, dissected DRGs were put in ice-cold HBSS containing CaCl_2_ and MgCl_2_ (Gibco Life Technologies, Carlsbad, CA, USA).

### 4.4. Primary DRG Cultures

Dissected DRGs were put immediately into ice-cold HBSS containing CaCl_2_ and MgCl_2_ (Gibco Life Technologies, Carlsbad, CA, USA). Then, DRGs were centrifuged for 3 min at 1000× *g*, and supernatant was carefully aspirated. Afterward, DRGs were treated with collagenase (500 U/mL; Sigma-Aldrich, St. Louis, MO, USA) and dispase (2.5 U/mL; Roche, Basel, Swiss). After incubating the DRGs for 75 min at 37 °C, DRGs were again centrifuged for 3 min at 1000× *g*, and DRG tissue pellet was washed twice with 10 mL neurobasal medium (Gibco Life Technologies) containing 1% (*v/v*) Pen-Strep (Gibco Life Technologies) and 10% (*v/v*) of inactivated FCS (Gibco Life Technologies). Between each washing step, DRGs were again centrifuged for 3 min at 1000× *g*. Before repeating the two washing steps, DRGs were treated for 10 min with 0.05% Trypsin-EDTA (Gibco Life Technologies) at 37 °C. At the end, supernatant was gently aspirated, and DRG tissue pellet was resuspended in 400 µL neurobasal medium (Gibco Life Technologies) containing 1% (*v/v*) Pen-Strep, 1% (*v/v*) L-glutamine (Gibco Life Technologies), 50 µg/mL (*v/v*) gentamicin (MP Biomedicals™, Santa Ana, CA, USA) and 1xB27-supplement (Gibco Life Technologies) and mechanically dissociated. Following this, 30 µL of dissociated DRGs was plated on poly-L-lysin (Sigma-Aldrich) coated glass coverslips. After incubating the plated DRGs at 37 °C for 2 h, 2 mL of neurobasal medium (+1% (*v/v*) Pen-Strep, 1% (*v/v*) L-glutamine, 50 µg/mL (*v/v*) gentamicin and 1xB27-supplement) was added to the DRGs. Then, DRGs were incubated overnight at 37 °C and 5% CO_2_ [55].

### 4.5. HEK293-Cell Cultivation

For HEK cell cultivation, DMEM (1×)+GlutaMAX™-I (Gibco Life Technologies, Carlsbad, CA, USA) containing 1% (*v/v*) Pen-Strep (Gibco Life Technologies) and 10% (*v/v*) of inactivated FCS (Gibco Life Technologies) was used. HEK cells were cultivated in 10 mm^2^ culture dishes at 37 °C and 5% CO_2_ humidified atmosphere.

### 4.6. HEK293 Cell Transfection

Ca^2+^ imaging in vitro experiments and PI(4,5)P2 ELISA measurement analysis were performed with transfected HEK cells as a heterologous expression system.

For Ca^2+^ imaging prior to HEK cell transfection with pEGFP-C1-(r)TRPM8, pCDNA3.1(+)GFP empty-vector (both vectors from Addgene, Watertown, MA, USA) or pCMV6-AC-GFP-(h)TRPV1 (OriGene Technologies, Rockville, MD, USA) HEK cells were plated with a confluency of 40% on poly-L-lysin coated glass coverslips (2 coverslips per 1× 6-well dish) and incubated at 37 °C for 2 h. For PIP_2_ ELISA measurement, HEK cells were first seeded with a confluency of 50% in a 6-well plate and then incubated at 37 °C for 2 h prior to HEK cell transfection with pEGFP-C1-(r)TRPM8 or pCDNA3.1(+)GFP empty-vector.

HEK cells were transfected as described previously [56]. Briefly, 1 µg of pEGFP-C1-(r)TRPM8, pCDNA3.1(+)GFP empty-vector or pCMV6-AC-GFP-(h)TRPV1 plasmid-DNA was added to 5 µL TurboFect™ (Thermo Fisher Scientific, Waltham, MA, USA) and 100 µL of serum free DMEM (1×)+GlutaMAX™-I (Gibco Life Technologies, Carlsbad, CA, USA). Afterward, the TurboFect™ and plasmid-DNA mixture was incubated for 20 min at RT for DNA-complex formation. At least 100 µL of the prepared transfection solution was carefully added dropwise to each 6-well dish or 6-well plate. Transfection efficiency was controlled by detecting the GFP signal of transfected HEK cells by use of an Axio Observer.Z1 microscope (Carl Zeiss, Oberkochen, Germany) after 24 h. Images were taken by using Zen 2.3 software (Carl Zeiss, Oberkochen, Germany).

### 4.7. HEK293 Cell Treatment

Prior to Ca^2+^ imaging measurement (r)TRPM8-transfected or empty-vector-transfected HEK cells were treated with 5 µM or 10 µM oxaliplatin or saline for 24 h. HEK cells transfected with (h)TRPV1 or empty-vector plasmid DNA were treated with 10 µM oxaliplatin prior to Ca^2+^ imaging experiments.

For identifying the related pathway that contribute to TRPM8 channel desensitization in (r)TRPM8-transfected HEK cells 24 h after oxaliplatin treatment, cells were treated with 10 µM oxaliplatin ±1 µM of the PLC inhibitor U 73122 (Tocris Bioscience, Bristol, UK), 10 µM oxaliplatin ±1 µM of the PKC inhibitor GF 109203X (Tocris) or 10 µM oxaliplatin ±10 µM or 25 µM of the PLC activator m-3M3FBS (Tocris) prior to Ca^2+^ imaging measurement.

For PI (4,5) P2 ELISA measurement analysis, (r)TRPM8-transfected or empty-vector-transfected and untransfected HEK cells were treated with 10 µM oxaliplatin for 24 h. For investigating the contribution of the PLC pathway to the PIP_2_ amount, (r)TRPM8-transfected or empty-vector-transfected and untransfected HEK cells were treated with 10 µM oxaliplatin ±10 µM of the PLC inhibitor D609 (Tocris) for 5 h.

### 4.8. Calcium Imaging

First, DRG neurons or transfected HEK cells were stained with Fura-2-AM (Biotium, Hayward, CA, USA) for 40 min. Afterward, DRG neurons or transfected HEK cells were washed twice with Ringer’s solution (pH 7.3) containing 145 mM NaCl, 1.25 mM CaCl_2_, 2 H_2_O, 1 mM MgCl_2_ 6 H_2_O, 5 mM KCl, 10 mM D-glucose (all purchased from Sigma-Aldrich, St. Louis, MO, USA) and 10 mM HEPES (AppliChem, Darmstadt, Germany). Baseline was recorded by flow through the DRG neurons or transfected HEK cells with Ringer’s solution [55].

For investigating the activity of the TRPM8, TRPA1, TRPV1, TRPM3, TRPV4 and P2X3 24 h or 1 h after oxaliplatin and vehicle treatment, DRG neurons of transfected HEK cells were stimulated with the ion channel specific agonists (TRPM8: 100 µM to 300 µM menthol (Sigma-Aldrich) for 30 s to 45 s; TRPA1: 100 µM allyl isothiocyanate (Sigma-Aldrich) for 30 s; TRPV1: 200 nM to 300 nM capsaicin (Merck KGaA, Darmstadt, Germany) for 20 s; TRPM3: 40 µM pregnenolone sulfate (Sigma-Aldrich) for 45 s; TRPV4: 1 µM GSK1016790A (Sigma-Aldrich) for 30 s; P2X3: 50 µM α,β-methylene ATP (Tocris, Bioscience, Bristol, UK) for 20s). DRG neurons were identified by stimulating the primary sensory neurons with 50 mM KCl for 60 s.

### 4.9. PI(4,5)P2 ELISA

To analyze the PI(4,5)P2 concentration, the (r)TRPM8-transfected, empty-vector-transfected and untransfected HEK cells, which were treated as previously described, were grown to 80% confluency in a 6-well plate. Afterward, the lipid extraction of PIP_2_ was performed according to the manufacturer´s instructions of the PIP_2_ ELISA kit (Echelon Biosciences, Salt Lake City, UT, USA) and as previously described [57]. Briefly, after carefully aspirating the old cell media, 1 mL of ice-cold 0.5 M TCA (Sigma-Aldrich, St. Louis, MO, USA) solution was added to the HEK cells. Next, HEK cells were immediately scraped from the 6-well plate. After centrifugation of the HEK cells for 7 min at 1000× *g* at 4 °C, the cell pellet was washed twice with 1 mL of a 5% TCA solution containing 1 mM EDTA (Honeywell Fluka™, Morristown, NJ, USA). Between each washing step, the HEK cells were first vortexed for 30 s and then centrifuged for 5 min at 1000× *g* at RT. Afterward, the supernatant was carefully aspirated, and the HEK cell pellet was resolved in 1 mL of MeOH:CHCl_3_ (2:1) (MeOH: Sigma-Aldrich; CHCl_3_: Fisher Scientific, Pittsburgh, PA, USA), then the mixture was vortexed for 10 min and centrifuged for 5 min at 1000× *g* at RT. This step was repeated one more time. Thereafter, 750 µL of MeOH:CHCl_3_:HCl (80:40:1) (HCl: Sigma-Aldrich) was added to the pellet. The mixture was vortexed for a further 25 min prior to centrifuging the mixture for a further 5 min at 1000× *g* at RT. Next, the supernatant was carefully transferred to a fresh 2 mL Eppendorf tube. Afterward, 250 µL of CHCl_3_ and then 450 µL of 0.1 N HCl were added to the supernatant. The mixture was vortexed for 30 s and then centrifuged for 5 min at 1000× *g* at RT. At least 500 µL of the lower organic phase was collected in a new 1.5 mL Eppendorf tube. Afterward, the lower organic phase was evaporated under a gentle stream of nitrogen at 45 °C. Extracted PIP_2_ lipid samples from HEK cells were stored at −20 °C until usage.

After PIP_2_ lipid extraction from the treated HEK cells, the PIP_2_ concentration was detected by performing a PIP_2_ ELISA according to manufacturer´s instructions and as previously described [58,59].

### 4.10. Quantitative Real-Time PCR

First, RNA from DRGs of oxaliplatin- and vehicle-treated male mice was isolated (24 h after treatment) by using the mirVana mRNA Isolation kit (Invitrogen by Thermo Fisher Scientific, Carlsbad, CA, USA). For cDNA syntheses, 400 ng of isolated RNA from DRGs was used. The cDNA syntheses were performed with the First Strand cDNA syntheses kit (Thermo Fisher Scientific, Waltham, MA, USA) following the manufacturer´s instructions. Finally, the following qPCR analysis of the TRPM3, TRPV4, kcnk2, kcnk4, hcn1, piezo1 and P2X3 transcript was performed by the use of assay primers of the TaqMan^®^ Gene Expression system (Thermo Fisher Scientific) on a QuantStudio 5 Real-Time PCR system (Thermo Fisher Scientific) according to manufacturer´s instructions and as previously described [60,61]. For relative mRNA expression quantification, the software QuantStudio™ Design and Analysis Software v1.4.3 (Thermo Fisher Scientific) was used. In addition, expression analysis was evaluated according to the ΔΔC(T) method, as described previously [62].

### 4.11. Data Analysis and Statitics

All data are represented as means ± SEM. For in vitro experiments comparing only two groups, the unpaired two-tailed *t*-test with Welch´s correction was used. For in vitro experiments comparing more than two groups, one-way analysis of variance (ANOVA) was used. Dunnett´s post hoc test was used for Ca^2+^ imaging and PI(4,5)P2 Elisa experiments; Sidak´s post hoc test was employed for qPCR analysis. Data with a *p*-value smaller than 0.05 were considered as statistically significant. Statistical analysis for all experiments was done by using GraphPad Prism 7 software (GraphPad, San Diego, CA, USA).

## 5. Conclusions

In summary, we showed that oxaliplatin leads to TRPM8 channel desensitization after transient TRPM8 channel activation and subsequent PLC pathway activation. Hence, transient changes in TRPM8 channel activity contribute to the induction of oxaliplatin-induced acute peripheral pain and may be targeted pharmacologically to reduce oxaliplatin-induced neuropathic pain.

## Data Availability

The data presented in this study are available on request from the corresponding author.

## Supplementary Materials

The following figures are available online at https://www.mdpi.com/article/10.3390/ijms22094962/s1.
