# Supplementary material for: Oxaliplatin Causes Transient Changes in TRPM8 Channel Activity
Source: Int J Mol Sci. 2021 May 7;22(9):4962. doi: 10.3390/ijms22094962 (PMC8125753; doi:10.3390/ijms22094962)
Supplement: Supplementary file 1 [file ijms-22-04962-s001.zip › ijms-1176363-supplementary.pdf]

Supplemental Information

Oxaliplatin Causes Transient Changes in TRPM8 Channel Activity

Vittoria Rimola [1], Tabea Osthues [2], Vanessa Königs [2], Gerd Geißlinger [1,2] and Marco Sisignano [1,2,*]

[1] Institute of Clinical Pharmacology, Pharmazentrum Frankfurt/ZAFES, University Hospital, Goethe-University, 60590 Frankfurt am Main, Germany; rimola@med.uni-frankfurt.de (V.R.); geisslinger@em.uni-frankfurt.de (G.G.)

[2] Fraunhofer Institute for Translational Medicine and Pharmacology ITMP, Theodor-Stern-Kai 7, 60596 Frankfurt am Main, Germany; tabea.osthues@itmp.fraunhofer.de (T.O.); Vanessa.Koenigs@itmp.fraunhofer.de (V.K.)

* Correspondence: marco.sisignano@med.uni-frankfurt.de; Tel.: +49-(0)69-6301-7819

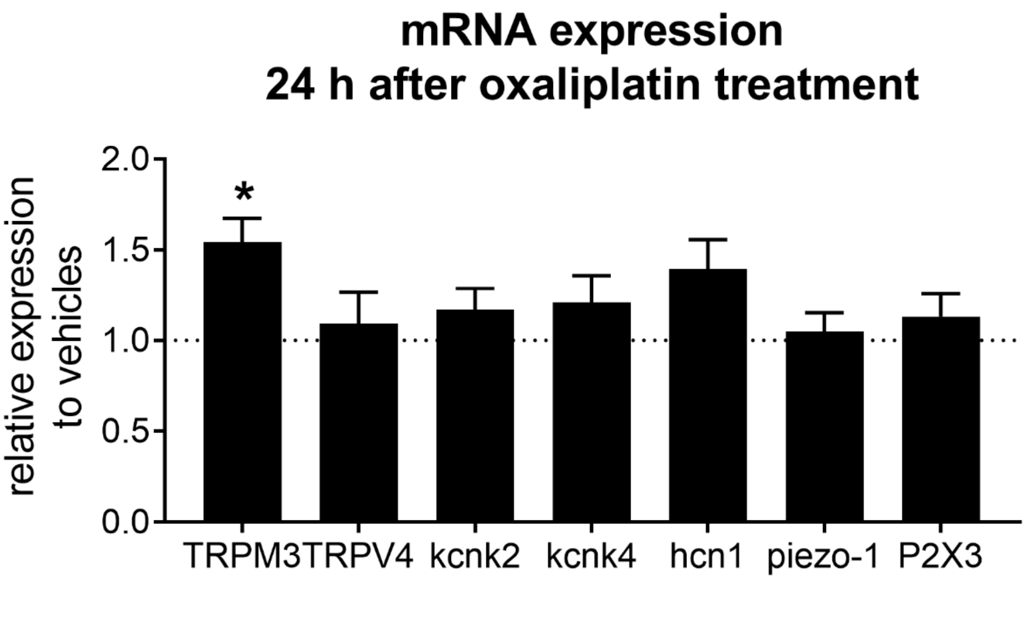

Figure S1. mRNA expression of different ion channels in DRGs 24 h after oxaliplatin or vehicle treatment. Relative mRNA expression of the TRPM3- (transient receptor potential melastatin 3), TRPV4- (transient receptor potential vanilloid 4), kcnk2- (two-pore domain potassium channel 2), kcnk4- (two-pore domain potassium channel 4), hcn1- (hyperpolarization-activated cyclic nucleotide-gated channel 1), piezo-1- and P2X3- (purinergic receptor 2X3) transcript. Data represents the means ± SEM from n=4-9 mice per condition; *$p<0.05$; one-way ANOVA and Dunnett´s multiple comparison test.

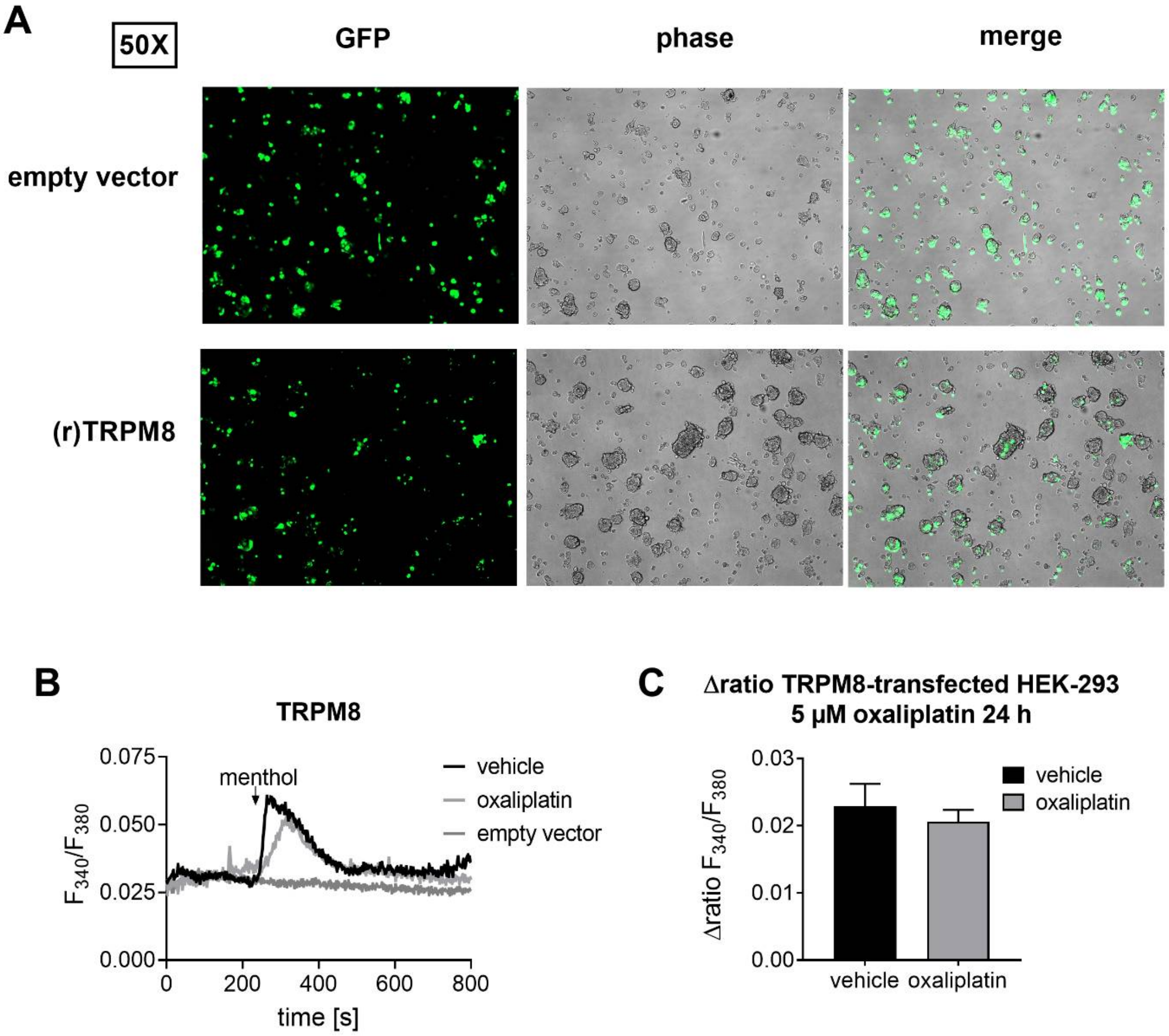

Figure S2. TRPM8-transfected HEK293-cells 24 h after oxaliplatin treatment (5 µM). (**A**) Representative fluorescence and phase contrast images of TRPM8 and empty-vector (pEGFP) transfected HEK293-cells captured at 50X magnification. (**B**) Representative Ca^{2+}-responses after menthol stimulation (300 µM, 45 s) of TRPM8 and empty-vector transfected HEK293-cells which were treated with vehicle (DMSO) or 5 µM oxaliplatin for 24 h. (**C**) Δratio F_{340}/F_{380} of the amplitude after a transient Ca^{2+}-influx after stimulating TRPM8-transfected HEK293-cells after oxaliplatin (5 µM) or vehicle treatment with menthol. Data represents the means ± SEM from n=39-44 TRPM8-transfected HEK293-cells per condition; two-tailed unpaired t-test with Welch´s correction.

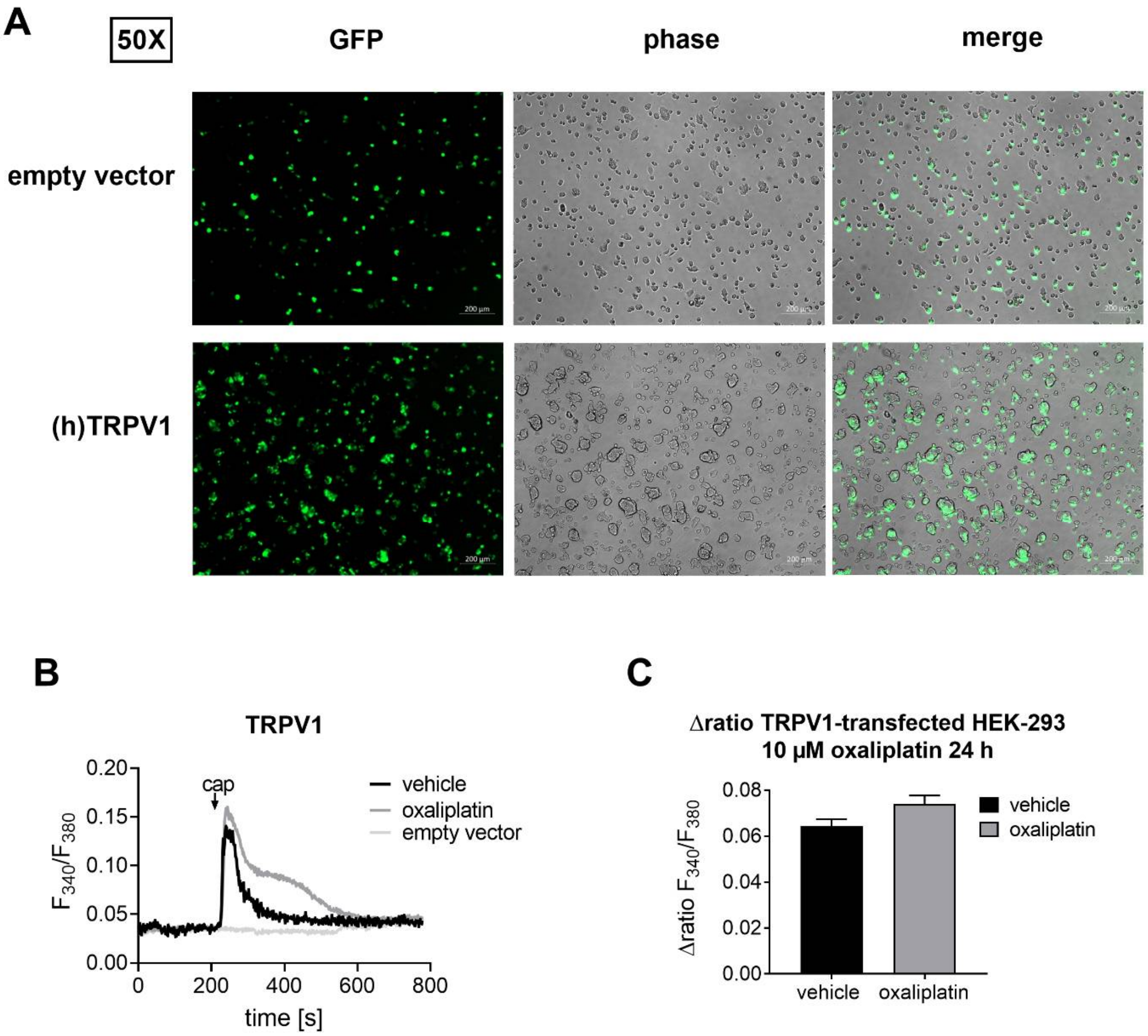

Figure S3. TRPV1-transfected HEK293-cells 24 h after oxaliplatin (10 µM) treatment. (**A**) Representative fluorescence and phase contrast images of TRPV1 and empty-vector (pEGFP) transfected HEK293-cells captured at 50X magnification. (**B**) Representative Ca^{2+}-responses after capsaicin (cap) stimulation (300 nM, 20 s) of TRPV1 and empty-vector transfected HEK293-cells which were treated with vehicle (DMSO) or 10 µM oxaliplatin for 24 h. (**C**) Δratio F_{340}/F_{380} of the amplitude after a transient Ca^{2+}-influx after stimulating TRPV1-transfected HEK293-cells after oxaliplatin (10 µM) or vehicle treatment with capsaicin. Data represents the means ± SEM from n=113-126 TRPV1-transfected HEK293-cells per condition; two-tailed unpaired t-test with Welch´s correction.
